# Smoking and drinking habits of women in subsequent pregnancies after specific advice about the dangers of these exposures during pregnancy

**DOI:** 10.7196/SAMJ.2020.v110i11.14667

**Published:** 2020-10-28

**Authors:** H J Odendaal, L T Brink, D G Nel, E Carstens, M Jager de, M Potter, C Plessis du, C A Groenewald

**Affiliations:** 1 Department of Obstetrics and Gynaecology, Faculty of Medicine and Health Sciences, Stellenbosch University, Cape Town, South Africa; 2 Department of Statistics and Actuarial Science, Faculty of Economic and Management Sciences, Stellenbosch University, Stellenbosch, South Africa

## Abstract

**Background.:**

Although women are informed about the dangers of drinking and smoking during pregnancy when they book for antenatal care, it is uncertain whether this advice is accepted, or whether attempts are made to apply it in subsequent pregnancies.

**Objectives.:**

To assess how pregnant women respond to the advice to refrain from smoking and drinking during pregnancy in subsequent pregnancies.

**Methods.:**

Research staff were trained to obtain accurate prospective information on smoking and drinking during pregnancy in a prospective study, using well-standardised methods. Care was taken to inform participants about the dangers of smoking and drinking during pregnancy. They were also given pamphlets on these dangers in their own language and a list of telephone numbers where they could find help to quit should they need it. This information was repeated at subsequent study visits (ranging from 1 to 3, depending on the gestational age at which they enrolled). Gestational age was determined by early ultrasound. *Z*-scores of birthweight for gestational age were determined according to the INTERGROWTH-21st study. Pregnancy outcomes of women who enrolled twice (*n*=888) or three times (*n*=77) in the Safe Passage Study were compared with those of women in the first enrolment (*n*=889).

**Results.:**

The proportion of drinkers did not change significantly (*p*=0.058) from the first to the second and third enrolments (63.8%, 59.0% and 54.6%, respectively). A similar trend was found for smokers (73.3%, 72.2% and 68.4%, respectively). Cannabis use was reported by 15.1%, 9.7% and 12.0% (*p*<0.005) of women, respectively, and use of methamphetamine by 10.1%, 6.6% and 12.7% (*p*<0.005). There was an increase in the rate of preterm births from 15.5% to 17.5% and 24.7%, respectively, but the increase was not significant. Although mean birthweight was lower in the third enrolment compared with the second, the difference was not significant. The *z*-score of birthweight for gestational age was significantly lower in the second enrolment compared with the first.

**Conclusions.:**

Detailed information on the adverse effects of smoking and drinking during pregnancy was not effective in the population studied. Other methods to reduce or stop these toxic exposures should therefore be investigated. A short inter-pregnancy interval, as demonstrated by three enrolments in 7.5 years, is associated with preterm labour and fetal growth restriction, and is probably indicative of the role played by confounders such as poor socioeconomic conditions and drug exposure during pregnancy.

Globally 72.5% of pregnant women are daily smokers, of whom 13.5% smoke heavily.^[1]^ Cigarette smoking and excessive maternal drinking during pregnancy are associated with increased numbers of stillbirths and sudden unexpected deaths.^[2,3]^ In addition, environmental exposure to cigarette smoke is associated with increased numbers of stillbirths and congenital abnormalities, and lower birthweights.^[4]^ Maternal alcohol intake prior to and during pregnancy is also associated with increased numbers of stillbirths and an increase in preterm labour and babies with lower birthweights.^[5,6]^

## Objectives

It is therefore essential to do as much as possible to reduce the risk of exposure to cigarette smoke and alcohol during pregnancy. During the Safe Passage Study (SPS) by the PASS (Perinatal Alcohol, SIDS, Stillbirth) Network, pregnant women were recruited at an antenatal clinic in the community where women at low risk for complications of pregnancy book for antenatal care.^[7]^ At enrolment in the study, the utmost care was taken to advise all participants about the adverse effects of smoking and/or drinking during pregnancy. As enrolment in the study extended over 7.5 years, many participants were recruited again during their next or even a subsequent pregnancy. Comparison of smoking and drinking habits between pregnancies could give an indication of the effectiveness of the information about the adverse effects of smoking and drinking provided during previous pregnancies. Smoking and drinking patterns during the first enrolment in the study were therefore compared with those during the second and third enrolments of the same participants.

## Methods

At recruitment, the purpose of the study and the need for informed consent were carefully explained to potential participants. Each participant received a signed copy of the consent form in Afrikaans or English (the two languages of the study community).

Research staff were specifically trained in the study procedures prior to initiation of the study, and throughout its course. Care was taken to inform participants about the dangers of smoking and drinking during pregnancy. They were also given pamphlets in their own language on these topics and a list of telephone numbers where they could find help to quit should they need it. This information was repeated at subsequent study visits (ranging from 1 to 3, depending on the gestational age at which they enrolled) and for all subsequent pregnancies.

Posters on smoking and drinking were displayed in the waiting room where participants waited before the study assessments. The same pamphlets were also placed on the notice boards of the assessment rooms. Information on the dangers of smoking was repeated by the research midwives at subsequent visits. Women with high Edinburgh depression scores, or social, drinking or drug-related problems, were referred to the social worker of the study, who advised further referrals to other departments, such as psychiatry, if necessary.

After completion of the study, the first study enrolment was compared with the second enrolment and a combination of the third and fourth (only one participant) visits. The inter-pregnancy intervals between the different enrolments were derived from the differences in dates of birth between enrolments.

Socioeconomic conditions, smoking and drinking patterns, neonatal biometry and the outcomes of pregnancy were then compared among the three enrolments.

Data were entered in Excel 365 (Microsoft Corp., USA) and exported to Statistica version 13 (TIBCO Software Inc., USA). Descriptive statistics were used to describe continuous variables, which were compared between groups with analysis of variance. The χ^2^ test determined significance in categorical data. Bonferroni or least significant difference multiple comparisons identified significant differences between the means. Spearman correlations measured correlations between repetitions of several response variables. Data with outliers were Winsorised by bringing the largest outliers and extremes closer to either the maximum or the minimum within 10% of the maximum or minimum of the data that were not outliers or extremes.

Permission to conduct this study was obtained from the Health Research Ethics Committee of Stellenbosch University (ref. no. N06/10/210) and the Western Cape Department of Health.

## Results

**Fig. 1.**
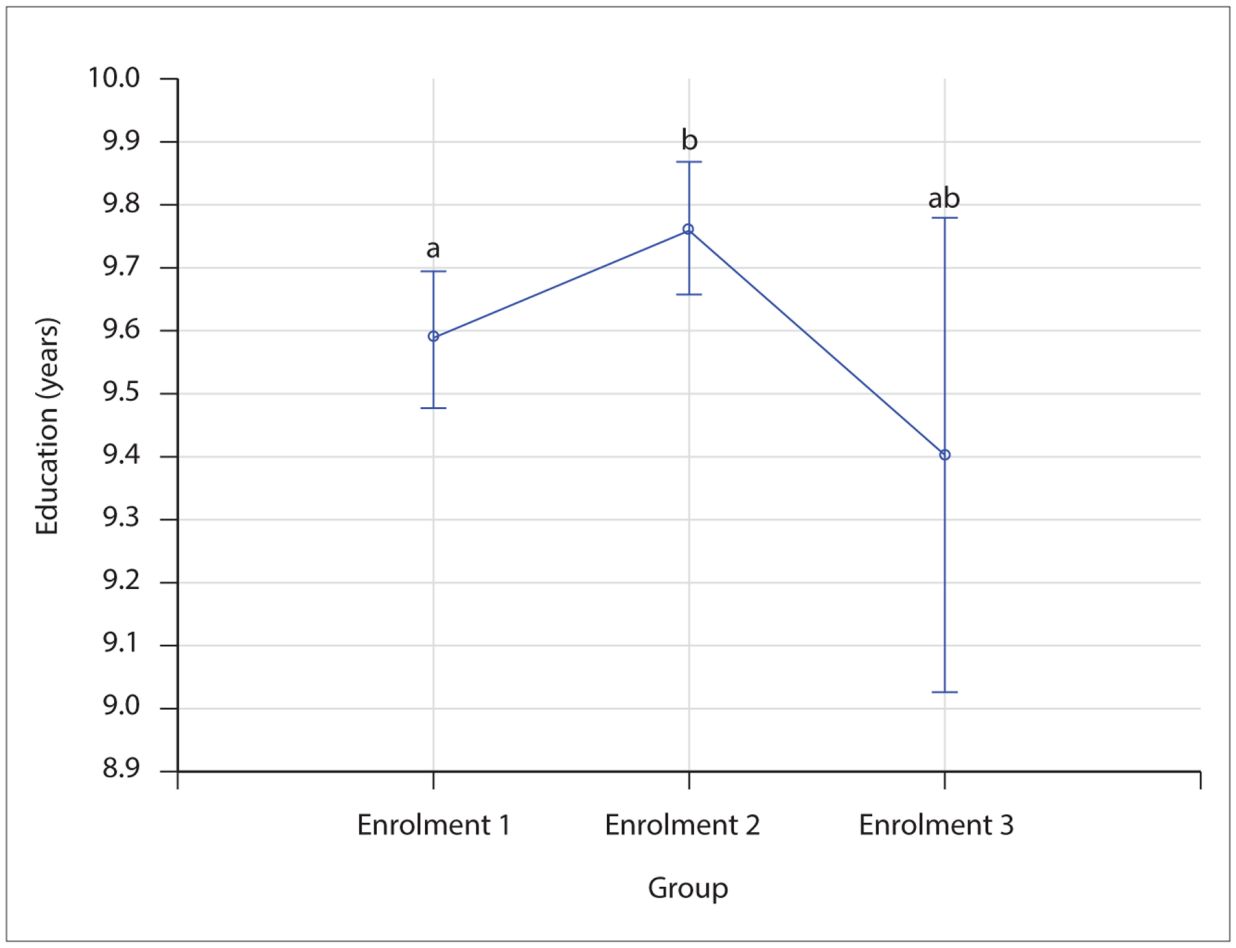
Significant differences between mean years of education for the different enrolments were found (*F*(2, 1 867)=3.3138; *p*=0.04). Whiskers denote 95% bootstrap confidence intervals, and duplicated letters above whiskers indicate absence of a significant difference.

**Fig. 2.**
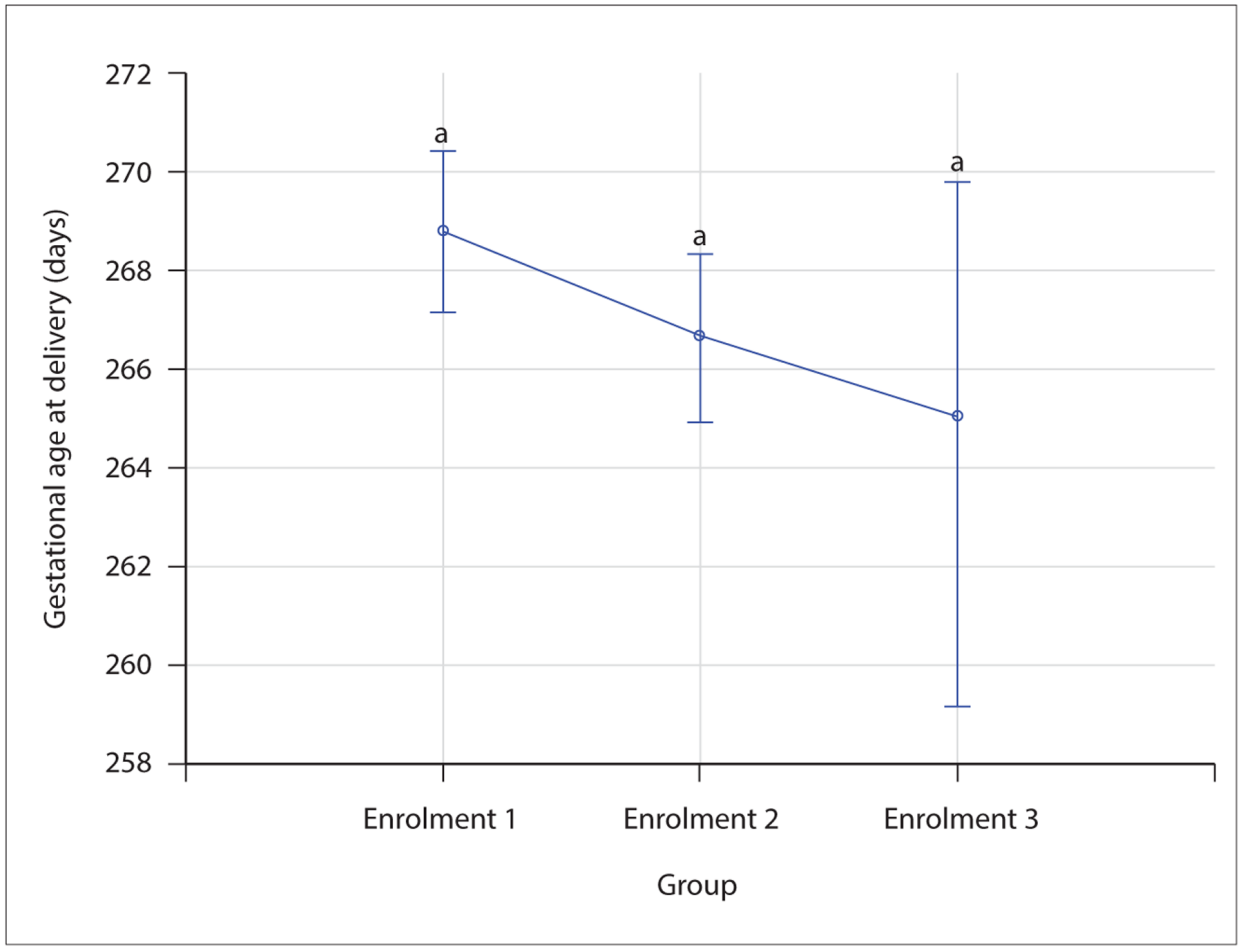
No significant differences between the gestational age at delivery means for the different enrolments were found (*F*(2, 1 868)=2.1024; *p*=0.12). Whiskers denote 95% bootstrap confidence intervals, and duplicated letters above whiskers indicate absence of a significant difference.

**Fig. 3.**
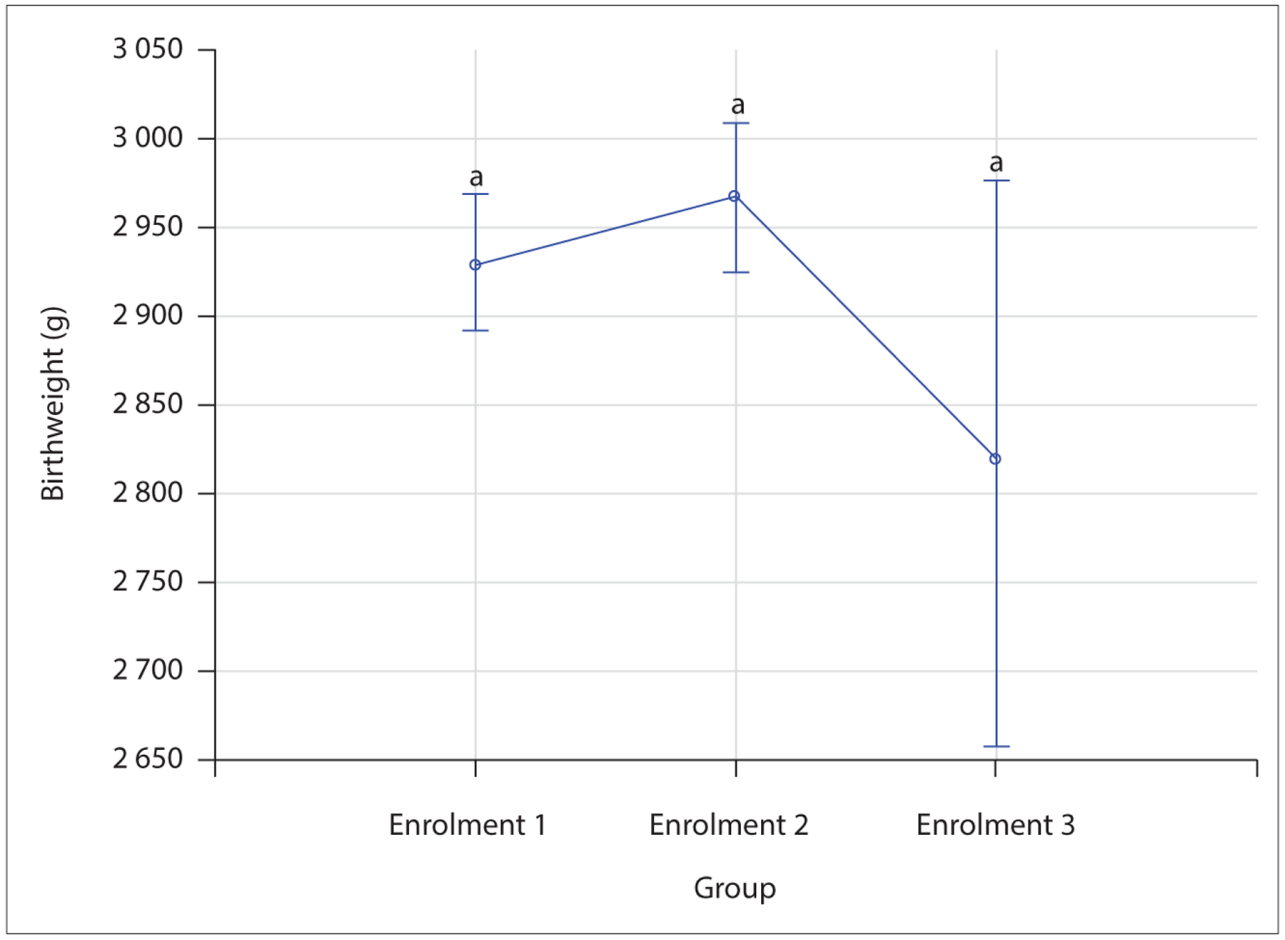
No significant differences between the birthweight means for the different enrolments were found (*F*(2, 1 868)=2.2486; *p*=0.09). Whiskers denote 95% bootstrap confidence intervals, and duplicated letters above whiskers indicate absence of a significant difference.

After exclusions of withdrawals and twin pregnancies from the 7 060 pregnant women recruited in the SPS study, 5 046 women were enrolled in the study only once and 1 854 women two to four times. There were 889 in the first enrolment, 888 (one woman was lost to follow-up) in the second and 77 in the third. The mean inter-pregnancy interval for women who were included in the study twice was 1 251 days. For women included three times, the mean intervals between the first two and last two pregnancies were 932 and 899 days, respectively. Table 1 reflects the comparison between the different enrolments. Maternal age differed significantly between the groups. When compared with enrolment 1, maternal body mass index was significantly higher in enrolment 2. When compared with enrolment 1, the number of years of formal education was significantly higher in enrolment 2. When compared with enrolment 1, mean household income was significantly lower in enrolment 2. The lowest duration of formal education was seen in enrolment 3, but the duration did not differ significantly from enrolments 1 and 2 (Fig. 1). When compared with enrolment 1, the mean Edinburgh depression score was significantly lower in enrolment 2. There were no significant differences between the three groups regarding total drinks during pregnancy, number of binge drinking episodes or mean number of cigarettes per day (Table 1). Although the gestational age at delivery declined after enrolment 1, it did not differ significantly between the three groups (Table 2, Fig. 2). When compared with enrolment 1, mean placental weight was significantly higher in enrolment 2. The same applied to the placental centile. Mean birthweight was lower in enrolment 3, but the differences between the three groups were not significant (Table 2, Fig. 3). Birthweight *z*-scores were significantly lower in enrolment 2 compared with enrolment 1 (Table 2).

In enrolment 1, 63.8% used alcohol compared with 59.0% and 54.6% in enrolments 2 and 3, respectively. The decline was not statistically significant (*p*=0.058) (Table 3). The proportion of smokers also did not change significantly between the groups, and remained around 68.4 – 73.7%. Use of cannabis declined in enrolment 2 but rose in enrolment 3. The highest proportion of methamphetamine users (17.3%) was observed in enrolment 3. The rate of preterm deliveries increased from 15.5% in enrolment 1 to 17.5% and 24.7% in enrolments 2 and 3, respectively, but the difference was not significant. No significant trends were found regarding the sex of the infant, miscarriages, terminations of pregnancy, stillbirths or neonatal and infant deaths.

## Discussion

We found that specific information on the adverse effects of smoking and drinking during pregnancy had little effect on limiting these exposures during subsequent pregnancies. Women who had three deliveries during the prospective study over 7.5 years seemed to be more at risk for poor perinatal outcome.

Low maternal income is associated with adverse fetal outcome. For example, in a study of mothers who had children diagnosed with fetal alcohol syndrome (FAS), the mean weekly income was ZAR818 per week, in comparison with ZAR2 406 per week in a randomly selected control group of 100 women.^[8]^ In a similar population, we found the lowest income in women experiencing their third pregnancy in the study.

We found that 54.6 – 63.8% of pregnant women in enrolments 1 – 3 (Table 3) used alcohol during pregnancy. A similar result (64.6%) was noted in the 5 046 women who were only enrolled in the study once (unpublished information, HJO; SPS), but these percentages are much higher than the 27.6% in a randomly selected control group in another study in a similar population, and even higher than the 50.8% of alcohol users in the 118 women who had children with FAS.^[8]^ In an earlier study, May *et al*.^[9]^ found that the prevalence of alcohol use was 24.2% in their control group.

We found that the prevalence rate of smokers ranged between 68.4% and 73.3% for the three enrolment groups (Table 3), which is similar to the 66.4% of women who were enrolled in the study only once (unpublished data, HJO; SPS). Our prevalence rate is similar to the 75% of women who had children diagnosed with FAS found by May *et al*.^[8]^ and much higher than the 32.7% of women in their control group.^[8]^ In another study, the prevalence rate of smoking in the control group of 133 women was 35.6%.^[9]^

In the multisite SPS, which comprised six different population groups,^[7]^ we found that 52.2% of women used alcohol during pregnancy and 47.9% smoked.

Our finding that 9.7 – 15.1% of women who had more than one delivery during the study period smoked cannabis during pregnancy was not much different from the cannabis use rate of 9.9% in women enrolled in the study only once (unpublished data, HJO; SPS), and similar to the prevalence rate of 9% we found in the cohort of 1 679 women in whom maternal serum alpha-fetoprotein (MSAFP) levels were determined.^[10]^

The same applies to the 6.6% of methamphetamine users found in enrolment 2 (Table 3) compared with 5% in women who were in the study once (unpublished data, HJO; SPS) or compared with 5% in women in whom MSAFP was measured.^[10]^ However, the highest rate of methamphetamine use (17.3%) was found in the group of 77 women who enrolled for their third pregnancy. The shorter pregnancy interval is likely to be the result of high-risk behaviour.

We found that birthweights were lower in enrolment 3 compared with enrolment 2, and *z*-scores for birthweight were significantly higher in enrolment 2 compared with enrolment 1. Although birthweights are usually higher with subsequent pregnancies in the general population,^[11]^ this was not seen in enrolment 3, possibly because of the shorter pregnancy interval and high-risk behaviour. Further analysis of the cohort of 5 938 women who registered for two pregnancies in the Collaborative Perinatal Project found that a short pregnancy interval is a primary marker for women who are otherwise at high risk, and that modification of this interval may be unlikely to have a major effect on low birthweight.^[12]^ This finding is supported by a more recent finding of a study using data on 1 416 women in the Scandinavian small-for-gestational-age study that the association between a short inter-pregnancy interval and low birthweight may reflect confounding by socioeconomic and other unmeasured factors.^[13]^

As some pregnant women have little knowledge of the consequences of tobacco use,^[14]^ several different programmes are available to facilitate quitting. These programmes include the implementation of system-wide complex healthcare intervention,^[15]^ incentives,^[16]^ self-help and clinical support,^[17]^ and integrated brief intervention.^[18]^ Schneider *et al*.^[19]^ did a systematic review of 19 identified studies and came to the conclusion that the rate of quitters varied from 4.0% to 69.7% for population-based studies and from 26.5% to 47.0% for clinical-based studies. Smoking cessation programmes during pregnancy seem to be cost-effective for preventing low birthweight if they cost <USD80 and they have achieved success rates of at least 18%.^[20]^ However, a Cochrane review found that there is insufficient evidence to show whether motivational intervention helps people to stop smoking compared with no intervention, as an addition to other types of behavioural support for smoking cessation, or compared with other types of behavioural support for smoking cessation.^[21]^

When compared with smoking, less information was available on intervention programmes to stop or reduce drinking during pregnancy. The C-BIAP (Computerized Brief Intervention for Alcohol use in Pregnancy) seems to be feasible and acceptable to pregnant women who do not report current drinking, and cognitive behavioural intervention seems to be helpful.^[22]^ This approach may be useful in clinics where staff time is limited.^[23]^ More recently, case management was evaluated in a population similar to the index study.^[24]^ It was demonstrated to be successful for women with high-risk drinking behaviour while pregnant. It is important to remember that more social support to quit smoking is associated with spontaneous alcohol abstinence.^[25]^

## Conclusions

Short inter-pregnancy intervals, as suggested by three enrolments in 7.5 years, are associated with preterm labour and growth restriction, and are therefore indicative of the probable role played by confounders such as poor socioeconomic conditions and cannabis and methamphetamine exposure during pregnancy. We have demonstrated that detailed information on the adverse effects of smoking and drinking during pregnancy was not effective in the population studied. Other methods to reduce harmful exposure should therefore be investigated.

## Figures and Tables

**Table 1.** Comparison of different enrolments*

	Enrolment 1	Enrolment 2	Enrolment 3
Maternal age (years)	^ac^22.9; 5.2; 22.5; 23.2	^ab^26.2; 5.0; 25.8; 26.5	^bc^27.7; 4.4; 26.6; 29.8
BMI (kg/m^2^)	^a^24.3; 5.2; 23.9; 24.7	^a^25.2; 5.9; 24.9; 25.6	25.0; 5.0; 23.8; 26.3
Gravidity	^ab^2.0; 1.8; 1.9; 2.1	^ac^3.1; 1.2; 3.0; 3.1	^bc^4.1; 1.3; 3.8; 4.3
Years of formal education	^a^9.6; 1.7; 9.5; 9.7	^a^9.8; 1.6; 9.6; 9.9	9.4; 1.7; 9.0; 7.8
Household income (ZAR/month)	^a^648; 500; 609; 688	^a^822; 495; 785; 859	706 ; 384 ; 583; 829
Edinburgh depression score	^a^13.8; 6.1; 13.4; 14.2	^a^12.6; 5.9; 12.2; 13.1	13.5; 6.1; 12.2; 13.1
Total standard drinks during pregnancy	22.4; 39.8; 18.9; 26.0	22.6; 46.3; 18.9; 26.3	28.9; 49.7; 15.8; 42.0
Total binges during pregnancy	2.3; 4.8; 2.1; 2.7	2.4; 5.1; 2.0; 2.9	3.1; 5.9; 2.0; 2.9
Cigarettes per day	4.3; 3.0; 4.0; 4.8	4.6; 4.0; 4.3; 4.9	4.3; 3.0; 3.2; 5.4
Gestational age at enrolment (days)	^a^147; 50; 144; 150	^a^136; 51; 133; 140	137; 56; 126; 149

BMI = body mass index; SD = standard deviation; LCL = lower confidence limit; UCL = upper confidence limit.

* Results are given as mean; SD; LCL; UCL, where LCL and UCL are the 95% lower and upper confidence limits, respectively.

a, b, c The same letter in different columns indicates significant differences between the means.

**Table 2.** Birth outcome in different enrolments*

	Enrolment 1	Enrolment 2	Enrolment 3
Gestational age at delivery (days)	269; 25; 267; 271	267; 26; 265; 268	265; 24; 259; 271
Placental weight (g)	^a^601; 142; 591; 612	^a^618; 146; 607; 628	620; 150; 585654
Placental weight centile	^a^39.1; 27.2; 37.1; 41.1	^a^42.9; 29.0; 40.9; 44.9	45.5; 27.7; 38.6; 52.3
Birthweight (g)	2 929; 592; 2 890; 2 969	2 966; 608; 2 926; 3 006	2 820; 668; 2 685; 2 955
Birthweight *z*-score	^a^−0.45; 1.0; −0.52; −0.39	^a^−0.27; 1.0; −0.34; −0.21	−0.38; 0.86; −0.61; −0.16

SD = standard deviation; LCL = lower confidence limit; UCL = upper confidence limit.

* Results are given as mean; SD; LCL; UCL, where LCL and UCL are the 95% lower and upper confidence limits, respectively.

a, b, c The same letter in different columns indicates significant differences between the means.

**Table 3.** Information on smoking, drinking, drug use and outcome of pregnancy*

	Enrolment 1 (*N*=889)^†^	Enrolment 2 (*N*=888)^†^	Enrolment 3 (*N*=77)^†^	χ^2^	*p*-value
Drinkers	567 (63.8)	524 (59.0)	42 (54.6)	5.7	0.058
Smokers	652 (73.7)	643 (72.3)	52 (68.4)	1.16	>0.05
Cannabis	129 (15.1)	85 (9.7)	9 (12.0)	11.53	<0.005
Methamphetamine	86 (10.1)	58 (6.6)	13 (17.3)	12.71	<0.005
Preterm deliveries	139 (15.5)	157 (17.5)	19 (24.7)	4.5	>0.05
Female infants	425 (48.0)	424 (48.0)	41 (53.3)	0.05	>0.05
Miscarriages	11 (1.23)	12 (1.34)	1 (1.30)	0.04	>0.05
Terminations of pregnancy	2 (0.23)	2 (0.23)	0	0.34	>0.05
Stillbirths	21 (2.38)	12 (1.36)	3 (3.95)	3.81	>0.05
Early neonatal deaths	5 (0.58)	6 (0.69)	1 (1.37)	0.53	>0.05
Late neonatal deaths	0	2 (0.23)	0	2.92	>0.05
Infant deaths in 1st year	14 (1.63)	6 (0.7)	1 (1.39)	3.41	>0.05

* Results given as *n* (%), e.g. for drinkers in enrolment 1: 567/889 = 63.8%, reported as 567 (63.8).

† The *N* may sometimes differ slightly in other variables when the specific information was not known.
